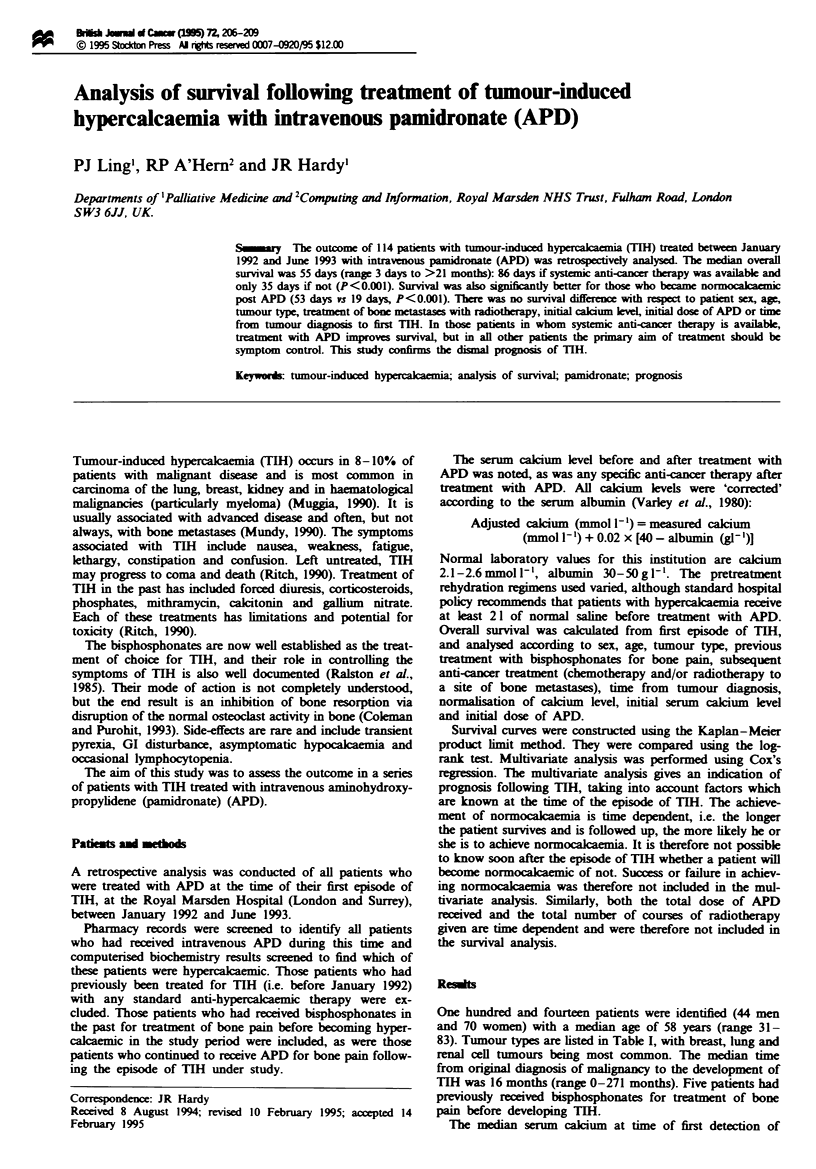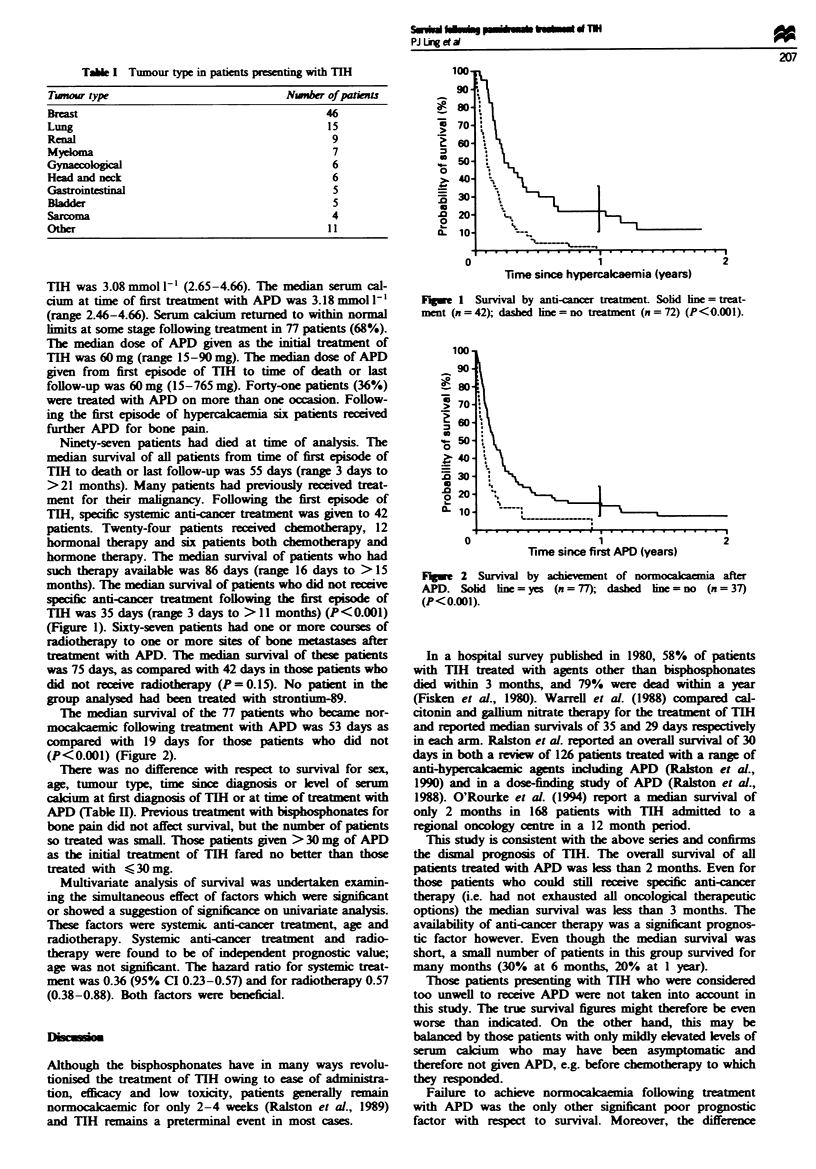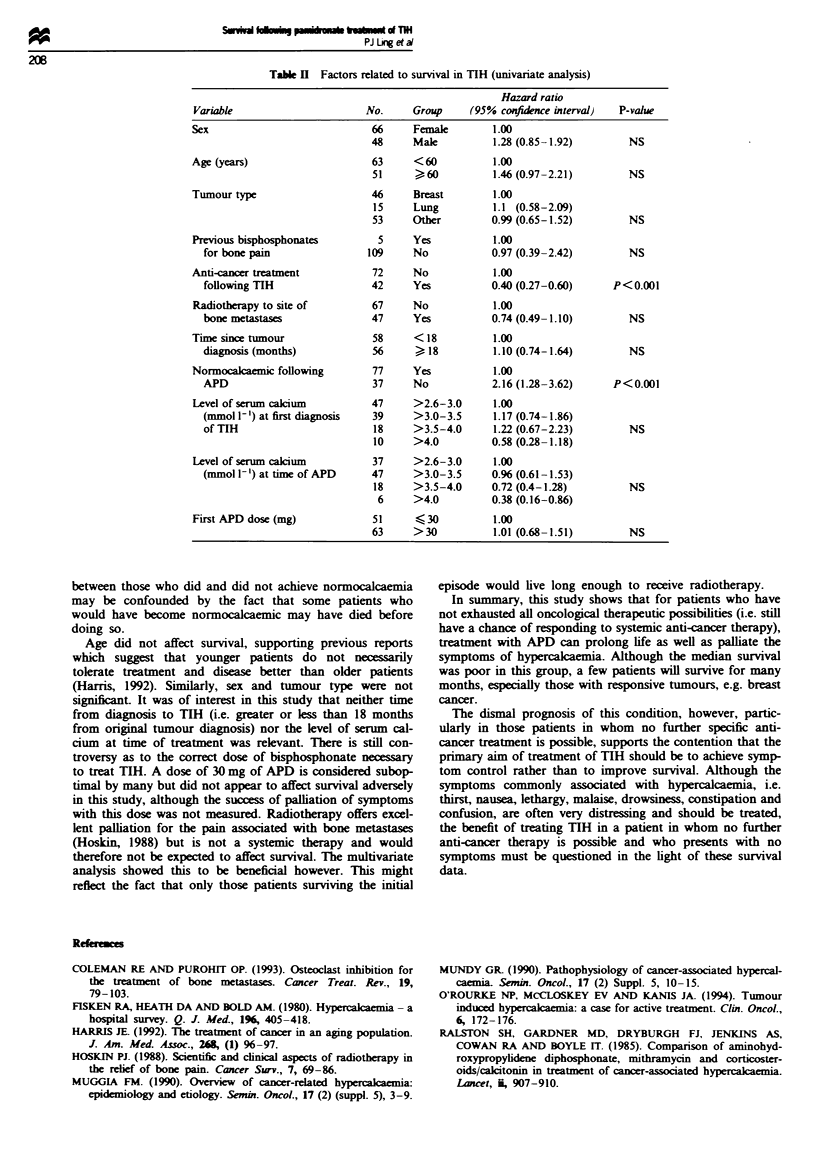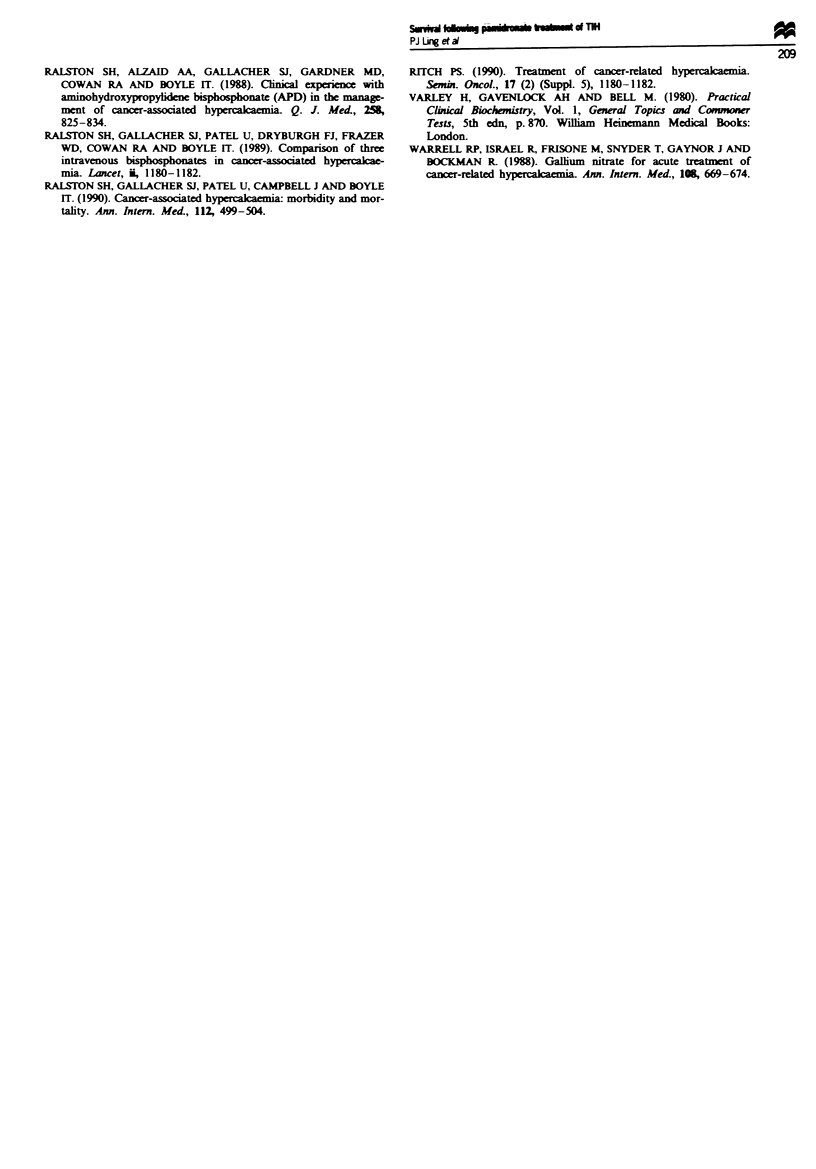# Analysis of survival following treatment of tumour-induced hypercalcaemia with intravenous pamidronate (APD).

**DOI:** 10.1038/bjc.1995.304

**Published:** 1995-07

**Authors:** P. J. Ling, R. P. A'Hern, J. R. Hardy

**Affiliations:** Department of Palliative Medicine, Royal Marsden NHS Trust, London, UK.

## Abstract

The outcome of 114 patients with tumour-induced hypercalcaemia (TIH) treated between January 1992 and June 1993 with intravenous pamidronate (APD) was retrospectively analysed. The median overall survival was 55 days (range 3 days to > 21 months): 86 days if systemic anti-cancer therapy was available and only 35 days if not (P < 0.001). Survival was also significantly better for those who became normocalcaemic post APD (53 days vs 19 days, P < 0.001). There was no survival difference with respect to patient sex, age, tumour type, treatment of bone metastases with radiotherapy, initial calcium level, initial dose of APD or time from tumour diagnosis to first TIH. In those patients in whom systemic anti-cancer therapy is available, treatment with APD improves survival, but in all other patients the primary aim of treatment should be symptom control. This study confirms the dismal prognosis of TIH.


### Analysis of survival following treatment of tumour-induced hypercalcaemia with intravenous pamidronate (APD)

PJ Ling[1], RP A'Hern[2] and JR Hardy[1]

*Departments of [1]Palliative Medicine and [2]Computing and Information, Royal Marsden NHS Trust, Fulham Road, London SW3 6JJ, UK.*

**Summary** The outcome of 114 patients with tumour-induced hypercalcaemia (TIH) treated between January 1992 and June 1993 with intravenous pamidronate (APD) was retrospectively analysed. The median overall survival was 55 days (range 3 days to >21 months): 86 days if systemic anti-cancer therapy was available and only 35 days if not ($P<0.001$). Survival was also significantly better for those who became normocalcaemic post APD (53 days *vs* 19 days, $P<0.001$). There was no survival difference with respect to patient sex, age, tumour type, treatment of bone metastases with radiotherapy, initial calcium level, initial dose of APD or time from tumour diagnosis to first TIH. In those patients in whom systemic anti-cancer therapy is available, treatment with APD improves survival, but in all other patients the primary aim of treatment should be symptom control. This study confirms the dismal prognosis of TIH.

**Keywords**: tumour-induced hypercalcaemia; analysis of survival; pamidronate; prognosis

Tumour-induced hypercalcaemia (TIH) occurs in 8–10% of patients with malignant disease and is most common in carcinoma of the lung, breast, kidney and in haematological malignancies (particularly myeloma) (Muggia, 1990). It is usually associated with advanced disease and often, but not always, with bone metastases (Mundy, 1990). The symptoms associated with TIH include nausea, weakness, fatigue, lethargy, constipation and confusion. Left untreated, TIH may progress to coma and death (Ritch, 1990). Treatment of TIH in the past has included forced diuresis, corticosteroids, phosphates, mithramycin, calcitonin and gallium nitrate. Each of these treatments has limitations and potential for toxicity (Ritch, 1990).

The bisphosphonates are now well established as the treatment of choice for TIH, and their role in controlling the symptoms of TIH is also well documented (Ralston *et al.*, 1985). Their mode of action is not completely understood, but the end result is an inhibition of bone resorption via disruption of the normal osteoclast activity in bone (Coleman and Purohit, 1993). Side-effects are rare and include transient pyrexia, GI disturbance, asymptomatic hypocalcaemia and occasional lymphocytopenia.

The aim of this study was to assess the outcome in a series of patients with TIH treated with intravenous aminohydroxypropylidene (pamidronate) (APD).

### Patients and methods

A retrospective analysis was conducted of all patients who were treated with APD at the time of their first episode of TIH, at the Royal Marsden Hospital (London and Surrey), between January 1992 and June 1993.

Pharmacy records were screened to identify all patients who had received intravenous APD during this time and computerised biochemistry results screened to find which of these patients were hypercalcaemic. Those patients who had previously been treated for TIH (i.e. before January 1992) with any standard anti-hypercalcaemic therapy were excluded. Those patients who had received bisphosphonates in the past for treatment of bone pain before becoming hypercalcaemic in the study period were included, as were those patients who continued to receive APD for bone pain following the episode of TIH under study.

Correspondence: JR Hardy
Received 8 August 1994; revised 10 February 1995; accepted 14 February 1995

The serum calcium level before and after treatment with APD was noted, as was any specific anti-cancer therapy after treatment with APD. All calcium levels were 'corrected' according to the serum albumin (Varley *et al.*, 1980):

$$\text{Adjusted calcium (mmol l}^{-1}) = \text{measured calcium (mmol l}^{-1}) + 0.02 \times [40 - \text{albumin (g l}^{-1})]$$

Normal laboratory values for this institution are calcium 2.1–2.6 mmol $l^{-1}$, albumin 30–50 g $l^{-1}$. The pretreatment rehydration regimens used varied, although standard hospital policy recommends that patients with hypercalcaemia receive at least 2 l of normal saline before treatment with APD. Overall survival was calculated from first episode of TIH, and analysed according to sex, age, tumour type, previous treatment with bisphosphonates for bone pain, subsequent anti-cancer treatment (chemotherapy and/or radiotherapy to a site of bone metastases), time from tumour diagnosis, normalisation of calcium level, initial serum calcium level and initial dose of APD.

Survival curves were constructed using the Kaplan–Meier product limit method. They were compared using the log-rank test. Multivariate analysis was performed using Cox's regression. The multivariate analysis gives an indication of prognosis following TIH, taking into account factors which are known at the time of the episode of TIH. The achievement of normocalcaemia is time dependent, i.e. the longer the patient survives and is followed up, the more likely he or she is to achieve normocalcaemia. It is therefore not possible to know soon after the episode of TIH whether a patient will become normocalcaemic of not. Success or failure in achieving normocalcaemia was therefore not included in the multivariate analysis. Similarly, both the total dose of APD received and the total number of courses of radiotherapy given are time dependent and were therefore not included in the survival analysis.

### Results

One hundred and fourteen patients were identified (44 men and 70 women) with a median age of 58 years (range 31–83). Tumour types are listed in Table I, with breast, lung and renal cell tumours being most common. The median time from original diagnosis of malignancy to the development of TIH was 16 months (range 0–271 months). Five patients had previously received bisphosphonates for treatment of bone pain before developing TIH.

The median serum calcium at time of first detection of

**Table I** Tumour type in patients presenting with TIH

| *Tumour type* | *Number of patients* |
|---|---|
| Breast | 46 |
| Lung | 15 |
| Renal | 9 |
| Myeloma | 7 |
| Gynaecological | 6 |
| Head and neck | 6 |
| Gastrointestinal | 5 |
| Bladder | 5 |
| Sarcoma | 4 |
| Other | 11 |

TIH was 3.08 mmol $l^{-1}$ (2.65–4.66). The median serum calcium at time of first treatment with APD was 3.18 mmol $l^{-1}$ (range 2.46–4.66). Serum calcium returned to within normal limits at some stage following treatment in 77 patients (68%). The median dose of APD given as the initial treatment of TIH was 60 mg (range 15–90 mg). The median dose of APD given from first episode of TIH to time of death or last follow-up was 60 mg (15–765 mg). Forty-one patients (36%) were treated with APD on more than one occasion. Following the first episode of hypercalcaemia six patients received further APD for bone pain.

Ninety-seven patients had died at time of analysis. The median survival of all patients from time of first episode of TIH to death or last follow-up was 55 days (range 3 days to >21 months). Many patients had previously received treatment for their malignancy. Following the first episode of TIH, specific systemic anti-cancer treatment was given to 42 patients. Twenty-four patients received chemotherapy, 12 hormonal therapy and six patients both chemotherapy and hormone therapy. The median survival of patients who had such therapy available was 86 days (range 16 days to >15 months). The median survival of patients who did not receive specific anti-cancer treatment following the first episode of TIH was 35 days (range 3 days to >11 months) ($P<0.001$) (Figure 1). Sixty-seven patients had one or more courses of radiotherapy to one or more sites of bone metastases after treatment with APD. The median survival of these patients was 75 days, as compared with 42 days in those patients who did not receive radiotherapy ($P=0.15$). No patient in the group analysed had been treated with strontium-89.

The median survival of the 77 patients who became normocalcaemic following treatment with APD was 53 days as compared with 19 days for those patients who did not ($P<0.001$) (Figure 2).

There was no difference with respect to survival for sex, age, tumour type, time since diagnosis or level of serum calcium at first diagnosis of TIH or at time of treatment with APD (Table II). Previous treatment with bisphosphonates for bone pain did not affect survival, but the number of patients so treated was small. Those patients given >30 mg of APD as the initial treatment of TIH fared no better than those treated with ⩽30 mg.

Multivariate analysis of survival was undertaken examining the simultaneous effect of factors which were significant or showed a suggestion of significance on univariate analysis. These factors were systemic anti-cancer treatment, age and radiotherapy. Systemic anti-cancer treatment and radiotherapy were found to be of independent prognostic value; age was not significant. The hazard ratio for systemic treatment was 0.36 (95% CI 0.23–0.57) and for radiotherapy 0.57 (0.38–0.88). Both factors were beneficial.

### Discussion

Although the bisphosphonates have in many ways revolutionised the treatment of TIH owing to ease of administration, efficacy and low toxicity, patients generally remain normocalcaemic for only 2–4 weeks (Ralston *et al.*, 1989) and TIH remains a preterminal event in most cases.

**Figure 1** Survival by anti-cancer treatment. Solid line = treatment ($n=42$); dashed line = no treatment ($n=72$) ($P<0.001$).

**Figure 2** Survival by achievement of normocalcaemia after APD. Solid line = yes ($n=77$); dashed line = no ($n=37$) ($P<0.001$).

In a hospital survey published in 1980, 58% of patients with TIH treated with agents other than bisphosphonates died within 3 months, and 79% were dead within a year (Fisken *et al.*, 1980). Warrell *et al.* (1988) compared calcitonin and gallium nitrate therapy for the treatment of TIH and reported median survivals of 35 and 29 days respectively in each arm. Ralston *et al.* reported an overall survival of 30 days in both a review of 126 patients treated with a range of anti-hypercalcaemic agents including APD (Ralston *et al.*, 1990) and in a dose-finding study of APD (Ralston *et al.*, 1988). O'Rourke *et al.* (1994) report a median survival of only 2 months in 168 patients with TIH admitted to a regional oncology centre in a 12 month period.

This study is consistent with the above series and confirms the dismal prognosis of TIH. The overall survival of all patients treated with APD was less than 2 months. Even for those patients who could still receive specific anti-cancer therapy (i.e. had not exhausted all oncological therapeutic options) the median survival was less than 3 months. The availability of anti-cancer therapy was a significant prognostic factor however. Even though the median survival was short, a small number of patients in this group survived for many months (30% at 6 months, 20% at 1 year).

Those patients presenting with TIH who were considered too unwell to receive APD were not taken into account in this study. The true survival figures might therefore be even worse than indicated. On the other hand, this may be balanced by those patients with only mildly elevated levels of serum calcium who may have been asymptomatic and therefore not given APD, e.g. before chemotherapy to which they responded.

Failure to achieve normocalcaemia following treatment with APD was the only other significant poor prognostic factor with respect to survival. Moreover, the difference

**Table II** Factors related to survival in TIH (univariate analysis)

| *Variable* | *No.* | *Group* | *Hazard ratio (95% confidence interval)* | P-*value* |
|---|---|---|---|---|
| Sex | 66 | Female | 1.00 | |
| | 48 | Male | 1.28 (0.85–1.92) | NS |
| Age (years) | 63 | <60 | 1.00 | |
| | 51 | ⩾60 | 1.46 (0.97–2.21) | NS |
| Tumour type | 46 | Breast | 1.00 | |
| | 15 | Lung | 1.1 (0.58–2.09) | |
| | 53 | Other | 0.99 (0.65–1.52) | NS |
| Previous bisphosphonates for bone pain | 5 | Yes | 1.00 | |
| | 109 | No | 0.97 (0.39–2.42) | NS |
| Anti-cancer treatment following TIH | 72 | No | 1.00 | |
| | 42 | Yes | 0.40 (0.27–0.60) | $P<0.001$ |
| Radiotherapy to site of bone metastases | 67 | No | 1.00 | |
| | 47 | Yes | 0.74 (0.49–1.10) | NS |
| Time since tumour diagnosis (months) | 58 | <18 | 1.00 | |
| | 56 | ⩾18 | 1.10 (0.74–1.64) | NS |
| Normocalcaemic following APD | 77 | Yes | 1.00 | |
| | 37 | No | 2.16 (1.28–3.62) | $P<0.001$ |
| Level of serum calcium (mmol $l^{-1}$) at first diagnosis of TIH | 47 | >2.6–3.0 | 1.00 | |
| | 39 | >3.0–3.5 | 1.17 (0.74–1.86) | |
| | 18 | >3.5–4.0 | 1.22 (0.67–2.23) | NS |
| | 10 | >4.0 | 0.58 (0.28–1.18) | |
| Level of serum calcium (mmol $l^{-1}$) at time of APD | 37 | >2.6–3.0 | 1.00 | |
| | 47 | >3.0–3.5 | 0.96 (0.61–1.53) | |
| | 18 | >3.5–4.0 | 0.72 (0.4–1.28) | NS |
| | 6 | >4.0 | 0.38 (0.16–0.86) | |
| First APD dose (mg) | 51 | ⩽30 | 1.00 | |
| | 63 | >30 | 1.01 (0.68–1.51) | NS |

between those who did and did not achieve normocalcaemia may be confounded by the fact that some patients who would have become normocalcaemic may have died before doing so.

Age did not affect survival, supporting previous reports which suggest that younger patients do not necessarily tolerate treatment and disease better than older patients (Harris, 1992). Similarly, sex and tumour type were not significant. It was of interest in this study that neither time from diagnosis to TIH (i.e. greater or less than 18 months from original tumour diagnosis) nor the level of serum calcium at time of treatment was relevant. There is still controversy as to the correct dose of bisphosphonate necessary to treat TIH. A dose of 30 mg of APD is considered suboptimal by many but did not appear to affect survival adversely in this study, although the success of palliation of symptoms with this dose was not measured. Radiotherapy offers excellent palliation for the pain associated with bone metastases (Hoskin, 1988) but is not a systemic therapy and would therefore not be expected to affect survival. The multivariate analysis showed this to be beneficial however. This might reflect the fact that only those patients surviving the initial episode would live long enough to receive radiotherapy.

In summary, this study shows that for patients who have not exhausted all oncological therapeutic possibilities (i.e. still have a chance of responding to systemic anti-cancer therapy), treatment with APD can prolong life as well as palliate the symptoms of hypercalcaemia. Although the median survival was poor in this group, a few patients will survive for many months, especially those with responsive tumours, e.g. breast cancer.

The dismal prognosis of this condition, however, particularly in those patients in whom no further specific anti-cancer treatment is possible, supports the contention that the primary aim of treatment of TIH should be to achieve symptom control rather than to improve survival. Although the symptoms commonly associated with hypercalcaemia, i.e. thirst, nausea, lethargy, malaise, drowsiness, constipation and confusion, are often very distressing and should be treated, the benefit of treating TIH in a patient in whom no further anti-cancer therapy is possible and who presents with no symptoms must be questioned in the light of these survival data.

### References

COLEMAN RE AND PUROHIT OP. (1993). Osteoclast inhibition for the treatment of bone metastases. *Cancer Treat. Rev.*, **19**, 79–103.

FISKEN RA, HEATH DA AND BOLD AM. (1980). Hypercalcaemia – a hospital survey. *Q. J. Med.*, **196**, 405–418.

HARRIS JE. (1992). The treatment of cancer in an aging population. *J. Am. Med. Assoc.*, **268**, **(1)** 96–97.

HOSKIN PJ. (1988). Scientific and clinical aspects of radiotherapy in the relief of bone pain. *Cancer Surv.*, **7**, 69–86.

MUGGIA FM. (1990). Overview of cancer-related hypercalcaemia: epidemiology and etiology. *Semin. Oncol.*, **17** (2) (suppl. 5), 3–9.

MUNDY GR. (1990). Pathophysiology of cancer-associated hypercalcaemia. *Semin. Oncol.*, **17** (2) Suppl. 5, 10–15.

O'ROURKE NP, MCCLOSKEY EV AND KANIS JA. (1994). Tumour induced hypercalcaemia: a case for active treatment. *Clin. Oncol.*, **6**, 172–176.

RALSTON SH, GARDNER MD, DRYBURGH FJ, JENKINS AS, COWAN RA AND BOYLE IT. (1985). Comparison of aminohydroxypropylidene diphosphonate, mithramycin and corticosteroids/calcitonin in treatment of cancer-associated hypercalcaemia. *Lancet*, **ii**, 907–910.

RALSTON SH, ALZAID AA, GALLACHER SJ, GARDNER MD, COWAN RA AND BOYLE IT. (1988). Clinical experience with aminohydroxypropylidene bisphosphonate (APD) in the management of cancer-associated hypercalcaemia. *Q. J. Med.*, **258**, 825-834.

RALSTON SH, GALLACHER SJ, PATEL U, DRYBURGH FJ, FRAZER WD, COWAN RA AND BOYLE IT. (1989). Comparison of three intravenous bisphosphonates in cancer-associated hypercalcaemia. *Lancet*, **ii**, 1180-1182.

RALSTON SH, GALLACHER SJ, PATEL U, CAMPBELL J AND BOYLE IT. (1990). Cancer-associated hypercalcaemia: morbidity and mortality. *Ann. Intern. Med.*, **112**, 499-504.

RITCH PS. (1990). Treatment of cancer-related hypercalcaemia. *Semin. Oncol.*, **17** (2) (Suppl. 5), 1180-1182.

VARLEY H, GAVENLOCK AH AND BELL M. (1980). *Practical Clinical Biochemistry*, Vol. 1, *General Topics and Commoner Tests*, 5th edn, p. 870. William Heinemann Medical Books: London.

WARRELL RP, ISRAEL R, FRISONE M, SNYDER T, GAYNOR J AND BOCKMAN R. (1988). Gallium nitrate for acute treatment of cancer-related hypercalcaemia. *Ann. Intern. Med.*, **108**, 669-674.